# The Impact of OXTR, COMT, and GRIN2B Polymorphisms on Brain Development in Preterm Infants

**DOI:** 10.3390/jcm14228233

**Published:** 2025-11-20

**Authors:** Eon Yak Kim, Hyuna Kim, Yong Hun Jang, Woochang Hwang, Junho K Hur, Young-Eun Kim, Sungmin Lim, Dong-Hye Ye, Hyun Ju Lee

**Affiliations:** 1 Department of Pediatrics, College of Medicine, Hanyang University, Seoul 04763, Republic of Korea; 2 Department of Pre-Medicine, College of Medicine, Hanyang University, Seoul 04763, Republic of Korea; 3 Hanyang Institute of Bioscience and Biotechnology, Hanyang University, Seoul 04763, Republic of Korea; 4 Department of Genetics, Graduate School of Medicine, Hanyang University, Seoul 04763, Republic of Korea; juhur@hanyang.ac.kr; 5 Department of Laboratory Medicine, College of Medicine, Hanyang University, Seoul 04763, Republic of Korea; 6 Department of Computer Science, Georgia State University, Atlanta, GA 30302, USA; dongye@gsu.edu; 7 Department of Translational Medicine, Graduate School of Biomedical Science and Engineering, Hanyang University, Seoul 04763, Republic of Korea; 8 Department of Biomedical Science, Graduate School of Biomedical Science and Engineering, Hanyang University, Seoul 04763, Republic of Korea; 9 Division of Neonatology and Developmental Medicine, Seoul Hanyang University Hospital, Seoul 04763, Republic of Korea; 10 Department of Pediatrics, Hanyang University Seoul Hospital, 222-1 Wangsimni-Ro, Seoul 04763, Republic of Korea

**Keywords:** *OXTR*, *COMT*, neurodevelopment, BSID-III, brain network, small-worldness, preterm infants

## Abstract

**Background/Objectives**: Preterm infants are at risk for developmental delays due to immature brain development and increased sensitivity to environmental stress. Genetic factors, such as polymorphisms in *OXTR* rs2268490, *COMT* rs4818, and *GRIN2B*, may influence these vulnerabilities and affect neurodevelopment. **Methods**: We recruited 91 preterm infants (<35 weeks gestation) admitted to the NICU at Hanyang University Seoul Hospital between January 2020 and December 2022. Brain MRIs were conducted at term-equivalent age, and DNA samples were analyzed for SNPs. Neurodevelopmental assessments were performed at 18 months corrected age using the Korean Developmental Screening Test (K-DST) and Bayley Scales of Infant Development, Third Edition (BSID-III). **Results**: Carriers of the minor alleles in *OXTR* rs2268490 showed significantly lower language and adaptive behavior, and *COMT* rs4818, rs740603 showed significantly lower social–emotional scores on BSID-III. *OXTR* rs2268490 was also associated with altered brain network metrics, including decreased small-worldness (*p* = 0.012) and increased global (*p* = 0.038) and local efficiency (*p* = 0.042). **Conclusions**: Polymorphisms in the *OXTR* genes are associated with differences in brain network organization and neurodevelopmental outcomes in preterm infants. These variants may influence how environmental factors affect early brain development, highlighting the importance of genetic screening and early intervention.

## 1. Introduction

Brain development in preterm infants is a critical factor that directly influences their quality of life and is considered a top priority following life-saving interventions. Infants born before term are exposed to various environmental stressors and immature organ functions, making it more difficult for their brains to develop in a stable environment than in full-term infants [1]. Despite comparable clinical exposures, preterm infants exhibit substantial differences in neurodevelopmental outcomes, indicating a potential role of genetic factors in shaping brain development [2,3,4,5].

Recent research indicates that several common single-nucleotide polymorphisms (SNPs), previously linked to neuropsychiatric disorders in adulthood, may exert their influence during early brain development by modulating neural circuit formation, synaptic plasticity, and neurotransmitter regulation [6,7,8]. These findings suggest that the roots of many adult-onset neuropsychiatric conditions may lie in the early stages of brain development when gene expression is initiated [8]. Among the candidate genes implicated in early neurodevelopmental processes, the oxytocin receptor (*OXTR*), catechol-O-methyltransferase (*COMT*), and NMDA receptor subunit 2B gene (*GRIN2B*) have garnered particular attention due to their pivotal roles in shaping neural circuitry. *OXTR* is involved in the modulation of social cognition and affective processing, with its polymorphisms repeatedly linked to atypical socioemotional development and a heightened susceptibility to ASD [9,10,11]. The *COMT* gene regulates dopamine metabolism in the prefrontal cortex, influencing mood regulation, cognition, and stress response, and its variants have been linked to psychiatric conditions such as ASD and schizophrenia [12,13]. In our previous study, we found that certain *OXTR* and *COMT* gene variants could influence cortical development in preterm infants at a term-equivalent age [14]. *GRIN2B* encodes the 2 B subunit of the NMDA receptor [15], which is crucial for attention regulation, memory, intelligence, and learning [16]. Numerous studies have reported an association between *GRIN2B* variants and attention-deficit/hyperactivity disorders (ADHD) [17,18]. Although such genetic associations have primarily been explored in children and adults, investigations of their roles in early brain development in preterm infants are still emerging. Given that the neonatal and infant periods are critical for brain development, research on how specific genetic variants influence brain development in preterm infants is highly warranted. To uncover these genotype–phenotype relationships, rigorous quantification of brain structure and function using advanced neuroimaging techniques is indispensable. Diffusion tensor imaging (DTI), a noninvasive quantitative neuroimaging technique, has gained attention for this purpose. DTI allows the assessment of not only macrostructural anatomy, but also microstructural architecture, such as anisotropy and fiber directionality, through tractography techniques [19]. More recently, the application of graph theory to DTI data has enabled researchers to characterize brain networks and assess network efficiency, allowing for the comparison of network alterations across different clinical conditions [20].

Neurodevelopmental outcomes in preterm infants are commonly assessed using standardized developmental evaluation tools. The Bayley Scales of Infant and Toddler Development, Third Edition (Bayley-III), is widely used to objectively measure cognitive, language, motor, and social development at early infancy [21]. A meta-analysis of studies in preterm populations demonstrated the strong predictive validity of the Bayley-III [22]. In Korea, prior to the administration of the BSID-III, a parent-reported screening tool, the Korean Developmental Screening Test for Infants and Children (K-DST), is used to identify children who may require further evaluation. If the K-DST indicates the need for more comprehensive assessment, tools such as BSID-III are employed by specialists. Although the K-DST is a screening tool, it has shown a high correlation with the BSID-III in studies involving preterm infants, suggesting its reliability [23,24]. However, few studies have investigated the relationship between SNP genotypes and neurodevelopmental outcomes in preterm infants in Korea. We previously explored the associations between certain SNP genotypes (*OXTR*, *COMT*, and *FADS2*) and K-DST outcomes, but we found no significant correlations [14]. Therefore, further studies using more comprehensive assessments such as the BSID-III are necessary to clarify these potential associations.

Integrating genomic information with neuroimaging and developmental assessments offers a powerful approach to unraveling the complex interplay between genetic variation, brain structure, and neurobehavioral outcomes. Despite the increasing recognition of such gene–brain–behavior associations, multidisciplinary investigations targeting common genetic variants in preterm populations remain limited, particularly within Korean cohorts. To address this gap, the present study conducted a genome-wide association analysis (GWAS) focusing on single-nucleotide polymorphisms (SNPs) in three genes—*OXTR*, *GRIN2B*, and *COMT*—all of which have been implicated in critical neurodevelopmental processes. We examined the associations between these SNPs, structural brain network characteristics derived from diffusion tensor imaging (DTI), and neurodevelopmental performance measured using the Bayley Scales of Infant and Toddler Development (BSID-III). Through this integrative approach, our aim was to elucidate the neurobiological mechanisms by which common genetic polymorphisms shape early brain development and to provide foundational data for personalized intervention strategies and early prognostic modeling in preterm infants.

## 2. Materials and Methods

### 2.1. Study Design and Participants

**Figure 1:**
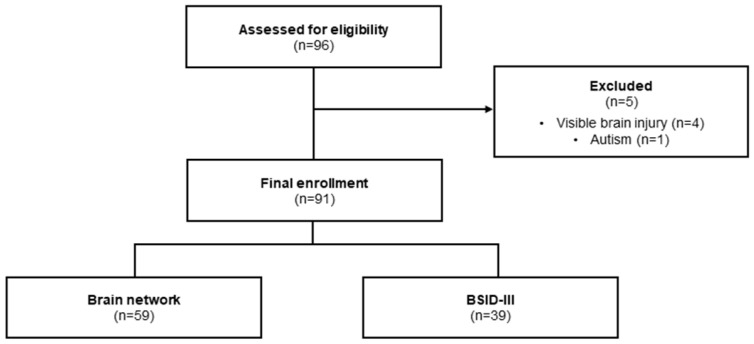
Flow diagram of this study. BSID, Bayley scales of infant and toddler development.

To mitigate the potential influence of ethnicity on population stratification, we included only patients of Korean descent in this study, which was performed in accordance with the ethical standards of the 1964 Declaration of Helsinki and the Institutional Review Board of Hanyang University Hospital (IRB no. 2018-12-022-025). This study assessed the brain MRI scans at term-equivalent age (TEA) for preterm infants (GA < 35 weeks) admitted to the neonatal intensive care unit at Hanyang University Seoul Hospital. Following our prior investigation [14], we prospectively expanded the study cohort and conducted longitudinal follow-up assessments to obtain comprehensive developmental outcome data. Infants born at less than 35 weeks of gestational age were included according to the inclusion criteria, and a total of 96 infants were admitted to the Hanyang Inclusive Clinic for Developmental Disorders at Hanyang University between December 2018 and December 2023. Written informed consent was obtained from the parents of all enrolled preterm infants. The exclusion criteria were major chromosomal anomalies (*n* = 0), congenital malformations (*n* = 0), intrauterine growth restriction (*n* = 0), evidence of visible brain injury (*n* = 4), and a documented family history of neurodevelopmental disorders (*n* = 1). Based on these criteria, a total of 5 infants were excluded from the final analysis—four with identifiable brain lesions on MRI, and one with a documented family history of autism spectrum disorder (ASD). A flowchart of this study is shown in Figure 1.

### 2.2. DNA Sample Collection

DNA samples were collected from participants after obtaining informed consent. Blood samples were processed, and DNA extracted using standard protocols (QIAsymphony DSP Circulating DNA Kit, Qiagen, Hilden, Germany). Genotyping was conducted using Illumina Infinium BeadChips, and SNP genotypes were derived using the BeadArrayFiles Python library version 3.0 (Detailed methods are described in Method S1). Single-nucleotide polymorphism (SNP) array analyses were conducted on DNA samples from 91 subjects. Each group was further categorized based on the presence of a minor allele, allowing for comparisons between minor homozygotes, heterozygotes, and major allele homozygotes. Based on previous studies, eight *OXTR* [9,14,25], *GRIN2B* [15,16,17,18], and *COMT* [12,13,14] SNPs were selected for analysis in this study, all of which exhibited significant deviations from the Hardy–Weinberg equilibrium (Table 1). Thus, eight SNPs were used to explore the association between brain volume, network, and genotype.

### 2.3. MRI Acquisitions

Preterm infants were scanned at near-term-equivalent age (35–44 weeks post-menstrual age, PMA) using a 3T MRI (Achieva, 16-channel phase-array head coil, Philips, Best, The Netherlands). All participants were scanned while sleeping naturally and physiological parameters were monitored during the MRI scans. In the DTI process, single-shot spin-echo three-dimensional (3D) echo-planar imaging sequences were used, with volume B0-shimming. Furthermore, T2-weighted images were captured for volumetric analysis, and white matter abnormalities were excluded. The specific parameters and detailed procedures used for acquiring MRI images are provided in Method S2.

### 2.4. Image Processing

**Figure 2:**
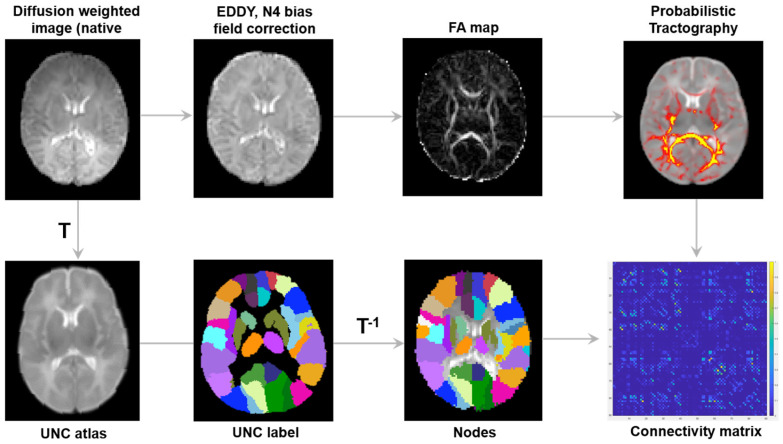
Overall flow chart of preprocessing for structural brain networks. FA, fractional anisotropy; T, transformation.

The processing pipeline for neonatal brain imaging, structural network reconstruction, and network topology analysis is depicted in Figure 2.

The DTI scans were processed using the FMRIB Software Library (FSL version 6.0.7.18, https://fsl.fmrib.ox.ac.uk/fsl/fslwiki (accessed on 5 September 2025)) [26]. Non-brain tissues were removed with the Brain Extraction Tool, and corrections for motion, eddy currents, and susceptibility-induced distortions were applied using eddy correction tool [27]. Low-frequency intensity inhomogeneities were corrected with N4 bias field correction in Advanced Normalization Tools (ANTs) [28], which was then applied to all diffusion-weighted images. Quality control involved a visual examination by two independent reviewers. Subsequently, the diffusion tensor model was established via simple least-squares fitting of the diffusion-weighted volumes, from which the principal eigenvalues were derived.

### 2.5. Network Construction

Structural brain networks were constructed by defining 90 brain regions (nodes) for each neonate. Individual b0 diffusion images were aligned with the University of North Carolina (UNC) neonate T2-weighted atlas using affine and nonlinear transformations [29], and atlas labels were mapped back to each participant’s native space. Nearest-neighbor interpolation was applied to preserve discrete anatomical labels. This procedure enabled consistent identification of brain regions across participants.

Edges of the network were generated using whole-brain probabilistic tractography with FSL (BEDPOSTX [30] and PROBTRACKX [31]), which modeled crossing fibers and corrected for partial volume effects. Connectivity between regions was quantified as the proportion of fibers connecting them, and bidirectional probabilities were averaged to form a symmetric 90 × 90 connectivity matrix.

Additionally, a pairwise Pearson correlation was conducted across all 4005 connections with nonzero probabilities among all participants, setting a correlation threshold (r = 0.7) to eliminate spurious connections with a low connection probability (Detailed methods are described in Method S3).

### 2.6. Global Network Analysis

Before quantifying the brain networks, a sparsity threshold of 0.25 was applied to individual structural networks to remove weak or spurious connections that were likely due to experimental noise rather than true anatomical connectivity [32,33]. The method used to select this threshold was in accordance with the procedure used in our previous network study [34,35].

Global network characteristics were examined using GRETNA software version 2.0.0 (Available Online: http://www.nitrc.org/projects/gretna/ (accessed on 12 September 2025)) [36]. To assess the global network structures, small-worldness (SW), global efficiency (GE), local efficiency (LE), and shortest path length (Lp) were employed [37]. To ensure robustness of the analysis, these global metrics were computed across 1000 random networks that maintained the same number of nodes, number of edges, and degree distributions.

### 2.7. Volumetric Analysis

Neonatal brain tissues were segmented and quantified from T2-weighted images using the morphologically adaptive neonatal tissue segmentation toolbox (MANTis) [38], which is specifically designed for neonates and improves upon conventional tissue classification methods. The brain was divided into eight tissue classes: gray matter, white matter, deep gray matter, hippocampus, amygdala, cerebellum, and brainstem. Tissue volumes were normalized by dividing by the total brain volume, excluding cerebrospinal fluid.

### 2.8. Neurodevelopmental Assessment

In this study, the Korean Developmental Screening Test for Newborns and Children (K-DST) was administered to preterm infants. Additionally, for the preterm infant group, the BSID-III was conducted to assess their developmental levels. The K-DST is a widely used assessment tool in Korea to evaluate the developmental milestones of infants and young children [39]. Tailored for the Korean cultural context and caregiving environment, the K-DST was developed and refined to identify potential developmental delays [24]. It comprises questionnaires covering six domains: gross motor function, fine motor function, cognition, language, social interaction, and self-help. Since self-help skills typically emerge after a certain developmental milestone, assessment in this domain begins at 18 months of age and was not included in this study. Each domain’s total score was categorized into one of four levels: higher than peer-level (≥1 SD), peer-level (<1 SD and ≥−1 SD), follow-up evaluation is recommended (<−1 SD and ≥−2 SD), and detailed evaluation is warranted (<−2 SD).The BSID-III is a standardized assessment tool used to measure the development of infants and toddlers [22]. Targeting ages from 1 to 42 months, it assesses cognitive, language, motor, social–emotional, and adaptive skills. The BSID-III provides standardized scores for each domain, with a mean of 100 and standard deviation of 15. These scores allow for comparison with the typical developmental milestones expected for a child’s age.

### 2.9. Statistical Analysis

A GWAS was utilized. To investigate the association between SNP and brain networks, correlation and regression analyses were performed, categorizing groups based on the presence or absence of minor alleles. The association between case–control status and each individual SNP was measured by the odds ratio and its corresponding 95% confidence interval using multiple logistic regression models adjusted for gestational age, sex, and brain network. Association Analyses were performed using the R software version 4.3.1 (http://www.r-project.org (accessed on 12 September 2024)). All analyses were performed assuming a dominant, recessive, codominant, additive, or allelic effect for each polymorphism. In the dominant model, heterozygous and rare homozygous variants were combined. In the recessive model, the variant was defined as only the rare homozygous genotype; in the codominant model, the variant was defined as the heterozygous variant or the rare homozygous variant; in the additive model, each genotype variant had the same effect; and in the allele model, the rare allele variant had an effect. The likelihood ratio test was used to test the effect of each SNP at a 5% significant level [40]. We controlled the false discovery rate (FDR) to address the multiple testing problems. Statistical analyses were conducted using SPSS (version 28.0; SPSS, Chicago, IL, USA), and the Benjamini and Hochberg method was used to control FDR (R version 4.3.1 (R Foundation for Statistical Computing, Vienna, Austria). with significance set at *p* < 0.05.

## 3. Results

We conducted a descriptive analysis of the demographic characteristics. The mean GA of the preterm group was 31.32 ± 3.68. Sixteen (17.6%) infants were affected by moderate-to-severe bronchopulmonary dysplasia (BPD). However, there were no differences in sex, stage II–III ROP, or developmental screening tests at 4 months of age. The demographic characteristics of the study cohort are summarized in Table 2. The mean ± SD of BSID-III, brain volume, and brain network are shown in Table S1.

### 3.1. Allelic and Genotypic Distributions

Among the SNPs in preterm infants included in the study, rs2268490 in *OXTR*, rs2268116 in *GRIN2B*, and rs740603 in *COMT* had higher frequencies of minor alleles than major alleles. The allele and genotype frequencies for all SNPs are presented in Table 3.

### 3.2. Association Between Minor Allele Frequencies and Neurodevelopmental Outcomes in Preterm Infants

The GWAS analysis investigating the relationship between MAF and neurodevelopmental assessments at 18 months revealed that individuals carrying the minor allele for the rs2268490 variant of *OXTR* exhibited a decrease of 6.51 points (*p* = 0.027) in language scores and 10.23 points (*p* = 0.026) in adaptive behavior scores compared to those carrying the major allele. Similarly, individuals with the minor allele for rs4818 of *COMT* showed a decrease of 12.25 points (*p* = 0.017) in social–emotional scores compared to those without, while those with the minor allele for rs740603 experienced a decrease of 11.55 points (*p* = 0.026). Details of the association between the MAF and BSID-III scores are shown in Table 4. However, there were no significant differences between the MAF and K-DST scores in the preterm group (Table S2) and in the full-term group for the *OXTR* gene only (Table S3).

### 3.3. Association Between Minor Allele Frequencies and Brain Network in Preterm Infants

After adjusting for age, sex, and postmenstrual age (PMA), which are factors that could influence brain network outcomes, association analysis revealed that individuals carrying the minor allele for rs1042778 of *OXTR* showed an increase in SW (B = 0.183, *p* = 0.009) and Lp (B = 2.101, *p* = 0.022) and a decrease in GE (B = −0.014, *p* = 0.030). Contrastingly, for rs2268493 of the *OXTR* gene, individuals with the minor allele exhibited lower SW (B = −0.145, *p* = 0.012) and Lp (B = −1.912, *p* = 0.010) but showed a trend toward higher GE (B = 0.011, *p* = 0.038) and LE (B = 0.018, *p* = 0.042). The adjusted model of the association between MAF and brain network metrics is summarized in Table 5.

### 3.4. Relationship Between Brain Quantitative Values and BSID-III Scores According to Allele Group in Preterm Infants

Among the 59 participants who underwent brain MRI and whose brain networks were successfully analyzed, a total of 39 infants completed the BSID-III assessment. The discrepancy between the number of participants who underwent MRI and those who completed the BSID-III was primarily attributable to non-cooperation during testing, as some infants were absent on the day of the developmental assessment or were unable to obtain valid scores due to limited attention span. No significant association was found between allele frequencies and volumetric variables in preterm infants (Table S4), whereas significant correlations were identified between certain network metrics and *OXTR*-rs1042778 and rs2268490.

**Figure 3:**
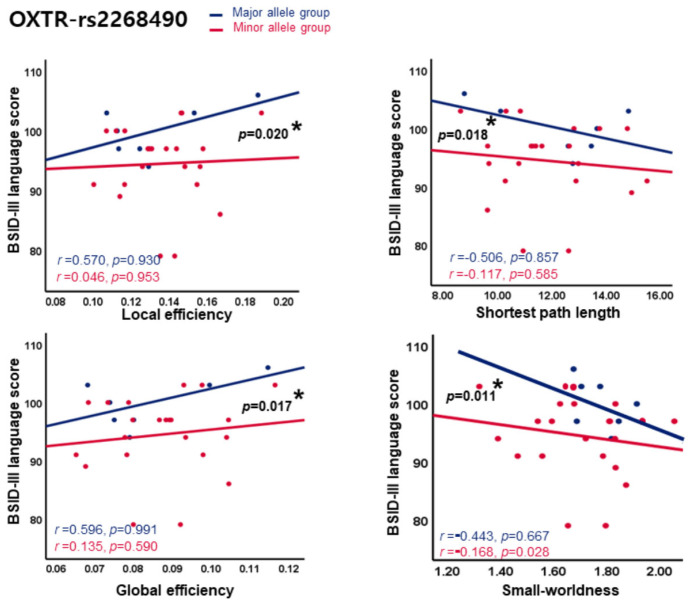
Interaction effect between network metrics and BSID-III score in *OXTR*-rs2268490. *OXTR*, oxytocin receptor; BSID, Bayley scales of infant and toddler development; SW, small-worldness; GE, global efficiency; LE, local efficiency; Lp, shortest path length. All *p*-value was adjusted for gestational age, sex, and postmenstrual age. * *p* < 0.05.

Nevertheless, in the case of *OXTR*-rs2268490, significant negative correlations were observed between SW and language scores exclusively within the minor allele group (*p* = 0.011) (Figure 3).

No significant relationships were found between the other network metrics and language scores in either group. Interestingly, significant differences in clinical characteristics were observed across the *OXTR* rs2268490 allele groups in the SW and GE (Tables S5 and S6), and the interaction between the groups revealed that the effect of each network metric on language scores differed depending on the allele group (all *p* < 0.05). Regarding COMPT-rs4818, no significant correlations were found between any network metric and social–emotional score. However, while there were no between-group differences in the clinical characteristics (Table S7), a significant interaction was noted only for Lp (*p* = 0.050).

## 4. Discussion

Guided by prior evidence implicating *OXTR*, *GRIN2B*, and *COMT* in neurodevelopmental processes and brain function, we selected these SNPs as candidate genetic markers for analysis. Previous studies have mainly examined these SNPs in relation to psychiatric and neurodevelopmental disorders during childhood, adolescence, or adulthood; therefore, research directly addressing their association with brain development in full-term infants is extremely limited. Nonetheless, a few noteworthy studies have been conducted in full-term populations [41,42,43]. For instance, one study on *OXTR* SNPs (although not targeting the same variants analyzed in our study) reported that *OXTR* rs1131149 could significantly influence social cognition in 18-month-old children [41]. However, our study did not yield significant findings in full-term infants, indicating that further research with larger cohorts is warranted to elucidate these associations.

Meanwhile, for these SNPs, GWAS was conducted in a cohort of preterm infants to investigate the relationships between these variants and neurodevelopmental outcomes, as well as structural brain features derived from diffusion tensor imaging (DTI)-based fiber tractography and standardized developmental assessments. No significant associations were observed between the SNP genotypes and outcomes in the K-DST. This result is consistent with the findings of our previous study [14]. However, using the BSID-III, we identified *OXTR* rs2268490, *COMT* rs4818, and *COMT* rs740603 as SNP genotypes that are significantly associated with developmental outcomes (Table 4).

The minor allele of *OXTR* rs2268490 was associated with lower scores in the language and adaptive behavior domains. Previous studies have reported associations between the minor allele of *OXTR* and ASD, including Asperger syndrome [44]. Other studies in adults have linked this variant to impairments in emotion recognition [45]. Among children and adolescents, genetic variations in *OXTR* have been shown to be associated with callous-unemotional (CU) traits, including conduct problems and antisocial behaviors [46]. Collectively, these studies suggest that *OXTR* rs2268490 is associated with impairments in communication and socioemotional understanding, which is in line with our finding of reduced neurodevelopmental scores in preterm infants carrying the minor allele of this SNP.

For *COMT*, both rs4818 and rs740603 were significantly associated with decreased socioemotional scores on the BSID-III. A pediatric study found that children carrying the rs4818 minor allele showed an increased prevalence of childhood-onset aggression (COA), diminished empathy, and CU traits characterized by abnormal processing of aversive emotional stimuli [47]. Additionally, in patients with schizophrenia, rs4818 was associated with negative symptoms, such as affective flattening and social withdrawal, as well as overall deficits in socio-emotional functioning [48]. Our prior work [14] identified an association between *COMT* rs740603 and decreased curvature of the left posterior cingulate, a brain region within the limbic lobe critical for self-referential thinking, autobiographical memory, and emotional reasoning. Given the role of this region, structural alterations may underlie the observed socioemotional impairments. Other studies have reported that rs740603 may contribute to negative symptoms in female patients with schizophrenia [49] and is associated with emotional impulsivity in individuals with depressive disorders carrying a minor allele [50]. In summary, our findings that *COMT* rs4818 and rs740603 are linked to socioemotional deficits in preterm infants are consistent with the existing literature.

Our results are significant for two reasons. First, to the best of our knowledge, this is the first study to directly investigate the association between *OXTR* rs2268490, *COMT* rs4818, and *COMT* rs740603 and neurodevelopmental outcomes in preterm infants. Second, our findings suggest that genetic factors known to affect neuropsychiatric outcomes in adulthood may begin to exert measurable effects on brain development during the neonatal period. Nevertheless, further studies are warranted to elucidate the precise mechanisms by which these SNPs influence neurodevelopment in preterm infants.

The lack of significant associations between the K-DST results and SNP genotypes may be attributable to the screening nature of the K-DST, which may not be sensitive enough to detect subtle neurodevelopmental changes associated with genetic variations. Although previous domestic studies in very low birth weight (VLBW) infants have reported strong correlations between the K-DST and BSID-III in the cognitive and language domains [23], the K-DST alone may not suffice for a comprehensive assessment. Given the limited number of studies investigating the association between common variant genes and neurodevelopmental outcomes in preterm infants, continued research is needed to build evidence and test the emerging hypotheses. Based on our findings, preterm infants harboring specific common variant genotypes may present as developmentally normal on the K-DST yet demonstrate deficits in more detailed evaluations, such as the BSID-III. This underscores the importance of ongoing surveillance and proactive developmental assessments beyond the initial screening.

No significant associations were identified between brain volume and SNP genotype. This aligns with previous studies investigating similar SNPs and their relationships with brain volume [14,51]. The literature on infant brain volume suggests that *OXTR*, *COMT*, and *GRIN2B* are unlikely to induce macroscopic changes during early infancy [52]. Therefore, we extended our investigation to microscopic brain structural connectivity using diffusion tensor tractography.

Among the examined SNPs, *OXTR* rs1042778 and rs2268490 were associated with brain network metrics. Notably, these two SNPs exhibited opposite trends in network topology. For *OXTR* rs1042778, increased SW and shortest path length (Lp) were accompanied by decreased GE, whereas rs2268490 showed the opposite pattern: decreased SW and shortest Lp with increased GE. To the best of our knowledge, no previous study has examined these specific genotypes in relation to structural brain network metrics derived from DTI in preterm infants. Most previous studies involving *OXTR* polymorphisms and brain connectivity have used functional MRI (fMRI) to assess functional rather than structural networks. However, Hermundstad et al. [53] reported a robust correspondence between structural and functional connectivity, allowing for inferences from fMRI-based findings.

For instance, Wang et al. [54] found that the minor allele of *OXTR* rs53576 was associated with decreased SW in a functional brain network due to an additive genetic effect. Although using a different SNP, this study highlights a trend wherein an increased minor allele load leads to a reduced SW. Similarly, Leanna et al. found that cumulative minor alleles across ASD-associated SNPs (rs53576, rs237887, rs1042778, and rs2254298) are associated with decreased within-connectivity in the brain, a measure of local clustering that supports localized processing [55]. As within-connectivity and SW are typically positively correlated, this body of literature supports our finding that the minor allele of *OXTR* rs2268490 is associated with reduced SW.

However, studies on *OXTR* rs1042778 have shown inconsistent results [56]. One study examining node centrality found regional variations in how the genotype affected network centrality, suggesting that the minor allele may not exert a uniform influence across the brain [10]. This may explain why rs1042778 and rs2268490 yielded opposite findings in our study and underscores the need for further research on the diverse regulatory effects of this SNP.

Perhaps the most compelling finding of this study is the identification of *OXTR* rs2268490 as an SNP linked to structural brain development and neurodevelopmental outcomes in preterm infants. We observed a significant negative correlation between SW and BSID-III language scores in the minor allele group and a notable interaction effect between the minor and major allele groups, supporting the notion that language-processing networks may be affected by excessive network segregation in the minor allele group. The SW reflects both functional segregation (modular specialization) and integration (efficient intermodule communication) in the brain [37,57,58]. While SW values above 1 are considered indicators of efficient networks, excessively high SW values may indicate oversegregation and compromised integration. One study on whole-brain structural organization in very preterm infants reported increased segregation and decreased integration, suggesting that excessive segregation may impede the developmental shift toward long-range integration [59]. This model aligns with our observation that increased SW is associated with lower language scores.

Additionally, the variability in SW and language scores was more pronounced in the minor allele group, whereas the major allele group showed a more stable distribution. This suggests that the minor allele of *OXTR* rs2268490 is associated with less stable and coherent patterns of brain development. In summary, preterm infants with the *OXTR* rs2268490 minor allele may struggle to achieve and maintain optimal levels of SW during brain development and excessive segregation may hinder the integration required for language processing, resulting in poor performance in the language domain.

To our knowledge, this is the first study to demonstrate that the *OXTR* rs2268490 polymorphism is associated with both structural brain network alterations and neurodevelopmental outcomes in preterm infants. Importantly, rs2268490 is located within an intronic region of the *OXTR* gene, a locus known to play a regulatory role in transcriptional activity and gene expression dynamics during early brain development [60]. Given the critical involvement of the *OXTR* gene in social cognition, language acquisition, and emotional regulation, it is plausible that the rs2268490 polymorphism may contribute to disruptions in these domains by altering transcriptional regulation and downstream gene expression patterns during early brain development. These findings highlight the dual influence of *OXTR* rs2268490 on brain structure and function, providing a rationale for future studies aimed at elucidating its underlying biological mechanism.

The limitations of this study include its focus on a single ethnic population (Korean), which may limit the generalizability of the findings. Further research in diverse populations is required. Additionally, the study was conducted using data from a single institution, raising concerns regarding sample size and selection bias. Further multicenter studies are required to validate and extend these findings.

## 5. Conclusions

In conclusion, our GWAS identified significant associations among common variant genes (*OXTR*, *GRIN2B*, and *COMT*), neurodevelopmental outcomes, and brain network metrics in preterm infants. *OXTR* and *COMT* variants were linked to lower scores in the language, adaptive, and socioemotional domains, whereas *OXTR* rs2268490 was significantly associated with structural brain network measures. Importantly, *OXTR* rs2268490 has emerged as a shared genetic factor influencing both neurodevelopment and brain connectivity, with the minor allele associated with disrupted and inconsistent brain development. The increased SW in this group was linked to decreased language scores, likely reflecting excessive segregation and impaired network integration. Our findings underscore the relevance of SNPs to both structural and functional aspects of brain development and provide a foundation for future research on the molecular mechanisms underlying these associations.

## Figures and Tables

**Table 1:** Allele frequencies, coding information, linkage disequilibrium details, and location of SNP.

Gene	SNP Name	Chromosome	Coordinate (Position)	Source	Variant (Major/Minor)	Korean MAF	Population MAF	HWE Unaffected, *p*
*OXTR*	rs1042778	3	8794545	dbSNP	G/T	0.087	0.076	0.504
*OXTR*	rs2268490	3	8797085	dbSNP	C/T	0.494	0.492	0.856
*OXTR*	rs2268493	3	8800840	1000_genomes	T/C	0.161	0.139	0.465
*GRIN2B*	rs2268116	12	13870080	1000_genomes	A/G	0.368	0.349	0.687
*GRIN2B*	rs2284411	12	13866172	dbSNP	C/T	0.186	0.164	0.736
*COMT*	rs174690	22	19939432	1000_genomes	G/A	0.283	0.290	0.824
*COMT*	rs4818	22	19951207	dbSNP	C/G	0.334	0.277	0.106
*COMT*	rs740603	22	19945177	dbSNP	A/G	0.423	0.357	0.548

SNP, single-nucleotide polymorphism; MAF, minor allele frequency; HWE, Hardy–Weinberg equilibrium; *OXTR*, oxytocin receptor; *GRIN2B*, glutamate ionotropic receptor N-methyl-D-aspartate type subunit 2 B; *COMT*, catechol-O-methyltransferase.

**Table 2:** Clinical characteristics of the cohort.

Characteristics	Preterm (*n* = 91)
Male sex	43 (47.3)
Gestational age, mean ± SD	31.32 ± 3.68
Birth weight, mean ± SD	1722.89 ± 698.96
Scan age, mean ±SD	37.95 ± 2.18 (59/91)
Moderate to severe BPD	16 (17.6)
Stage II to III ROP	6 (6.6)
K-DST	
<−2 SD in any domain	10/87 (11.5)
<−2 SD in gross motor domain	4/87 (4.6)
<−2 SD in fine motor domain	2/87 (2.3)
<−2 SD in cognition domain	5/87 (5.7)
<−2 SD in language domain	5/87 (5.7)
<−2 SD in sociality domain	1/87 (1.2)

Data are expressed as the mean ± SD or *n* (%). SD, standard deviation; BPD, bronchopulmonary dysplasia; ROP, retinopathy of prematurity; K-DST, Korean Developmental Assessment of Infants.

**Table 3:** Allelic and genotypic distributions of *OXTR*, *GRIN2B*, and *COMT* variants in preterm infants.

Gene	SNP Name	Variant	Preterm (*n* = 91)
*OXTR*	rs1042778	G/G	76 (84.4)
		G/T	13 (14.4)
		T/T	1 (1.1)
*OXTR*	rs2268490	T/T	21 (23.1)
		C/T	49 (53.8)
		C/C	21 (23.1)
*OXTR*	rs2268493	T/T	67 (73.6)
		T/C	24 (26.4)
		C/C	0 (0.0)
*GRIN2B*	rs2268116	A/A	41 (45.1)
		A/G	41 (45.1)
		G/G	9 (9.9)
*GRIN2B*	rs2284411	C/C	65 (71.4)
		C/T	24 (26.4)
		T/T	2 (2.2)
*COMT*	rs174690	G/G	47 (51.6)
		G/A	36 (39.6)
		A/A	8 (8.8)
*COMT*	rs4818	C/C	49 (53.8)
		C/G	34 (37.4)
		G/G	8 (8.8)
*COMT*	rs740603	A/A	39 (42.9)
		A/G	40 (44.0)
		G/G	12 (13.2)

SNP, single-nucleotide polymorphism; *OXTR*, oxytocin receptor; *GRIN2B*, glutamate ionotropic receptor N-methyl-d-aspartate type subunit 2 B; *COMT*, catechol-O-methyltransferase.

**Table 4:** Association of allele frequencies with neurodevelopment in preterm infants.

		BSID-III (*n* = 39)									
Gene	SNP Name	Cognition		Language		Motor		Social–Emotional		Adaptive Behavior	
		B (95% CI)	*p*	B (95% CI)	*p*	B (95% CI)	*p*	B (95% CI)	*p*	B (95% CI)	*p*
*OXTR*	rs1042778	−5.881 (−13.25 to 1.488)	0.125	−2.05 (−8.939 to 4.839)	0.563	−2.643 (−12.17 to 6.883)	0.589	−0.842 (−13.97 to 12.28)	0.900	7.171 (−3.501 to 17.84)	0.194
*OXTR*	rs2268490	−2.605 (−9.212 to 4.002)	0.444	−6.51 (−12.1 to −0.924)	0.027	−4.03 (−12.2 to 4.139)	0.339	−1.446 (−12.87 to 9.982)	0.805	−10.23 (−18.95 to −1.515)	0.026
*OXTR*	rs2268493	−2.073 (−8.215 to 4.069)	0.512	−2.455 (−7.874 to 2.964)	0.379	−0.197 (−7.852 to 7.459)	0.960	−2.566 (−13.15 to 8.021)	0.637	−1.675 (−10.2 to 6.846)	0.702
*GRIN2B*	rs2268116	0.121 (−6.017 to 6.258)	0.970	1.205 (−4.219 to 6.63)	0.665	−3.544 (−11.09 to 4.004)	0.362	−0.083 (−10.64 to 10.47)	0.988	−1.371 (−9.853 to 7.11)	0.753
*GRIN2B*	rs2284411	−0.862 (−7.34 to 5.616)	0.795	2.396 (−3.303 to 8.095)	0.414	−2.652 (−10.66 to 5.355)	0.519	2.338 (−8.791 to 13.47)	0.682	4.966 (−3.888 to 13.82)	0.277
*COMT*	rs174690	2.583 (−3.372 to 8.539)	0.400	0.043 (−5.272 to 5.357)	0.988	5.654 (−1.615 to 12.92)	0.134	6.722 (−3.419 to 16.86)	0.200	0.232 (−8.069 to 8.534)	0.957
*COMT*	rs4818	4.516 (−1.333 to 10.36)	0.137	4.032 (−1.145 to 9.209)	0.134	3.679 (−3.677 to 11.04)	0.332	−12.25 (−21.94 to −2.568)	0.017	−3.309 (−11.54 to 4.922)	0.435
*COMT*	rs740603	3.836 (−2.084 to 9.756)	0.210	−0.086 (−5.418 to 5.246)	0.975	−4.197 (−11.57 to 3.177)	0.270	−11.55 (−21.36 to −1.734)	0.026	−7.07 (−15.15 to 1.009)	0.093

BSID, Bayley scale of infant and toddler development; SNP, single-nucleotide polymorphism; *OXTR*, oxytocin receptor; *GRIN2B*, glutamate ionotropic receptor N-methyl-D-aspartate type subunit 2 B; *COMT*, catechol-O-methyltransferase. B is the unstandardized beta. Adjusted for gestational age and sex.

**Table 5:** Association of allele frequencies with brain networks in preterm infants.

		Brain Network (*n* = 59)							
Gene	SNP Name	SW		GE		LE		L_P_	
		B (95% CI)	*p*	B (95% CI)	*p*	B (95% CI)	*p*	B (95% CI)	*p*
*OXTR*	rs1042778	0.183 (0.050 to 0.316)	0.009	−0.014 (−0.027 to −0.002)	0.030	−0.020 (−0.041 to 0.000)	0.058	2.101 (0.360 to 3.842)	0.022
*OXTR*	rs2268490	−0.145 (−0.254 to −0.035)	0.012	0.011 (−0.016 to 0.004)	0.038	0.018 (0.001 to 0.034)	0.042	−1.912 (−3.331 to 0.512)	0.010
*OXTR*	rs2268493	−0.061 (−0.167 to 0.0451)	0.264	−0.006 (0.001 to 0.022)	0.241	−0.010 (−0.026 to 0.006)	0.226	0.747 (−0.622 to 2.116)	0.290
*GRIN2B*	rs2268116	0.010 (−0.093 to 0.112)	0.854	−0.000 (−0.010 to 0.009)	0.942	−0.008 (−0.019 to 0.012)	0.664	0.187 (−1.129 to 1.502)	0.782
*GRIN2B*	rs2284411	0.052 (−0.055 to 0.160)	0.342	−0.005 (−0.015 to 0.005)	0.369	−0.003 (−0.024 to 0.008)	0.308	0.557 (−0.827 to 1.941)	0.434
*COMT*	rs174690	−0.046 (−0.148 to 0.056)	0.380	0.004 (−0.005 to 0.014)	0.386	0.008 (−0.008 to 0.023)	0.334	−0.824 (−2.128 to 0.481)	0.221
*COMT*	rs4818	−0.005 (−0.107 to 0.097)	0.923	−0.002 (−0.011 to 0.008)	0.699	−0.002 (−0.017 to 0.013)	0.785	0.495 (−0.815 to 1.806)	0.462
*COMT*	rs740603	−0.084 (−0.183 to 0.016)	0.105	0.004 (−0.006 to 0.013)	0.426	0.006 (−0.009 to 0.021)	0.459	−0.226 (−1.536 to 1.084)	0.737

SNP, single-nucleotide polymorphism; *OXTR*, oxytocin receptor; *GRIN2B*, Glutamate ionotropic receptor N-methyl-D-aspartate type subunit 2 B; *COMT*, catechol-o-methyltransferase; SW, small world; GE, global efficiency; LE, local efficiency; Lp, local path length; PMA, postmenstrual age. B is the unstandardized beta. Adjusted for gestational age, sex, and PMA.

## Data Availability

The datasets generated and/or analyzed during the current study are available from the corresponding author upon reasonable request.

## Supplementary Materials

The following supporting information can be downloaded at: https://www.mdpi.com/article/10.3390/jcm14228233/s1, Method S1: DNA extraction and genotyping; Method S2: MRI acquisition details; Method S3: Network construction; Table S1: Neurodevelopmental outcome and brain volume and network in preterm infants; Table S2: Adjusted model of K-DST in preterm group; Table S3: Associations between OXTR SNPs and K-DST developmental domains in the full-term group; Table S4: Association of allele frequencies with brain volume in preterm infants; Table S5: Clinical characteristics of minor allele frequency of rs2268490 in OXTR; Table S6: Simple effect analysis of network metrics on language score by OXTR rs2268490 allele group; Table S7: Clinical characteristics of minor allele frequency of rs4818 in COMT.
