# Supplementary material for: The Impact of OXTR, COMT, and GRIN2B Polymorphisms on Brain Development in Preterm Infants
Source: J Clin Med. 2025 Nov 20;14(22):8233. doi: 10.3390/jcm14228233 (PMC12653483; doi:10.3390/jcm14228233)
Supplement: Supplementary file 1 [file jcm-14-08233-s001.zip › jcm-3950067-Supplementary Methods.pdf]

Supplementary Methods

Supplementary Method 1. *DNA extraction and genotyping*

After obtaining informed consent, venous blood samples were collected and stored following standardized biospecimen protocols to ensure sample integrity. Genomic DNA was extracted using the QIAsymphony DSP Circulating DNA Kit (cat. 937556; Qiagen, Hilden, Germany) in accordance with the manufacturer's instructions.

Following hybridization, the Illumina Infinium BeadChips were processed for the single-base extension reaction, staining, and imaging on an Illumina iScan scanner. Signal intensities were normalized, and genotypes were calculated using the Illumina BeadArrayFiles Python library, which converts fluorescence intensity data into SNP genotypes.

Each SNP was independently analyzed to identify clusters and genotype calls by comparing the observed signal distribution with reference cluster files (*.egt) provided by Illumina, generated using over 200 HapMap reference DNA samples.

Supplementary Method 2. *MRI acquisition details*

All MRI scans were conducted at near-term-equivalent age (35–44 weeks postmenstrual age) using a 3.0 Tesla Philips Achieva scanner equipped with a 16-channel phased-array head coil. Infants were scanned during natural sleep, swaddled in blankets to maintain temperature, and continuously monitored by a pediatrician for respiratory rate, heart rate, and oxygen saturation.

Diffusion tensor imaging (DTI) was acquired using single-shot spin-echo echo-planar imaging with the following parameters: TR = 4800 ms, TE = 75 ms, flip angle = 90°, Slice thickness = 2 mm, voxel size = 1.56 × 1.56 mm^2, Field of view (FOV) = 120 × 120 mm, 15 diffusion directions, b-value = 800 s/mm^2, 2 averages, Total scan duration = 6 min 17 s.

T2-weighted images were obtained for volumetric and structural analysis using a fast spin-echo sequence with: TR = 4800 ms, TE = 90 ms, flip angle = 90°, FOV = 180 × 180 mm^2, voxel size = 0.5 × 0.5 mm^2, Slice thickness = 3 mm, 1 average, total time = 6 min 30 s.

Infants with visible white matter injury or hemorrhage on T2-weighted images were excluded from analysis.

Supplementary Method 3. *Network construction*

Structural networks were constructed by defining 90 brain regions (nodes) for each neonate. First, individual b0 diffusion images were aligned to the T2-weighted images of the UNC neonate atlas using a 12-parameter affine transformation followed by a nonlinear symmetric normalization algorithm (ANTs). To maintain consistency, the automated anatomical labeling atlas from the UNC space was mapped back to each participant's native space using inverse transformations. Discrete anatomical labels were preserved using nearest-neighbor interpolation, which ensures that each voxel retains a single, distinct region assignment. This procedure enabled consistent identification of 90 brain regions across all participants, which served as the nodes of the structural network.

Whole-brain fiber tracking was performed using probabilistic tractography with FSL. BEDPOSTX was first applied to model crossing fibers and correct for partial volume effects in thick slices (settings: fiber=3, Rician noise distribution). Probabilistic tractography was then performed using PROBTRACKX on individual diffusion images. For each pair of regions i and j, the connectivity probability was defined as the proportion of fibers originating from all voxels in region i that reached all voxels in region j (PROBTRACKX parameters: 5,000 samples per seed voxel, step length = 0.5 mm, curvature threshold = 0.2, FA threshold = 0.01).

Since probabilistic tractography is direction-dependent, unidirectional probabilities (P_{ij}) (from i to j) may differ from (P_{ij}). A symmetric 90 × 90 connectivity matrix was created by averaging bidirectional probabilities. To reduce spurious connections, a pairwise Pearson correlation was calculated across all 4,005 nonzero connections among participants, and connections with correlation below 0.7 were removed. The remaining probabilities were used as weighted network edges ($W_{ij} = P_{ij}$) for subsequent network analysis.
