# Supplementary material for: The Impact of OXTR, COMT, and GRIN2B Polymorphisms on Brain Development in Preterm Infants
Source: J Clin Med. 2025 Nov 20;14(22):8233. doi: 10.3390/jcm14228233 (PMC12653483; doi:10.3390/jcm14228233)
Supplement: Supplementary file 1 [file jcm-14-08233-s001.zip › jcm-3950067_Supplementary Tables.pdf]

Supplementary Table 1. Neurodevelopmental outcome and brain volme and network in preterm infants

	**Preterm (n=91)**
**BSID-III**	(n=39)
Cognition	102.05±8.09
Language	96.36±6.23
Motor	102.54±12.21
Social-Emotional	105.26±19.73
Adaptive behavior	104.28±14.51
**Brain volume**	(n=59)
White matter	36.12±3.91
Grey matter	47.51±4.76
Deep grey matter	6.97±1.04
Hippocampus	0.85±0.65
Amygdala	0.54±1.05
Cerebellum	6.45±0.67
Brainstem	1.57±0.27
**Brain network**	(n=57)
SW	1.66±0.19
GE	0.09±0.02
LE	0.14±0.03
Lp	11.75±2.50

SD, standard deviation; BSID, Bayley scales of infant & toddler development ;SW, small-world; GE, global efficiency; LE, local efficiency; Lp, local path length.

Supplementary Table 2. Adjusted model of K-DST in preterm group

Gene	SNP Name	K-DST* (n=87)											
		**Any domain**		**Gross motor**		**Fine motor**		**Cognitive**		**Language**		**Sociality**	
		**Odd ratio (95% CI)**	***p***	**Odd ratio (95% CI)**	***p***	**Odd ratio (95% CI)**	***p***	**Odd ratio (95% CI)**	***p***	**Odd ratio (95% CI)**	***p***	**Odd ratio (95% CI)**	***p***
OXTR	rs1042778	0.913 (0.277 to 3.011)	0.881	1.331 (0.322 to 5.506)	0.693	0.618 (0.156 to 2.440)	0.492	1.457 (0.276 to 7.691)	0.657	1.045 (0.253 to 4.314)	0.952	3.619 (0.413 to 31.68)	0.245
OXTR	rs2268490	1.488 (0.548 to 4.039)	0.435	0.886 (0.279 to 2.813)	0.838	0.823 (0.229 to 2.959)	0.766	1.156 (0.322 to 4.156)	0.824	1.058 (0.331 to 3.381)	0.924	1.331 (0.366 to 4.841)	0.664
OXTR	rs2268493	0.556 (0.206 to 1.501)	0.246	0.603 (0.203 to 1.789)	0.362	1.855 (0.467 to 7.375)	0.380	0.896 (0.249 to 3.225)	0.867	1.469 (0.428 to 5.041)	0.541	0.846 (0.164 to 1.940)	0.364
GRIN2B	rs2268116	0.603 (0.247 to 1.468)	0.603	0.812 (0.300 to 2.198)	0.682	1.229 (0.419 to 3.599)	0.707	0.362 (0.101 to 1.294)	0.118	0.634 (0.224 to 1.796)	0.391	0.648 (0.194 to 2.171)	0.482
GRIN2B	rs2284411	0.795 (0.306 to 2.066)	0.638	0.822 (0.283 to 2.390)	0.719	0.507 (0.163 to 1.583)	0.242	0.732 (0.217 to 2.467)	0.615	0.521 (0.180 to 1.506)	0.229	0.923 (0.255 to 3.339)	0.923
COMT	rs174690	0.734 (0.297 to 1.815)	0.503	0.673 (0.242 to 1.869)	0.447	1.172 (0.384 to 3.575)	0.780	0.690 (0.213 to 2.236)	0.536	0.913 (0.320 to 2.601)	0.865	1.054 (0.314 to 3.536)	0.932
COMT	rs4818	0.948 (0.396 to 2.269)	0.904	0.908 (0.339 to 2.427)	0.847	0.516 (0.173 to 1.534)	0.234	0.543 (0.172 to 1.717)	0.299	0.859 (0.312 to 2.368)	0.770	0.835 (0.261 to 2.674)	0.761
COMT	rs740603	1.163 (0.482 to 2.803)	0.737	0.787 (0.288 to 2.154)	0.641	0.696 (0.229 to 2.118)	0.523	1.378 (0.444 to 4.274)	0.578	0.900 (0.322 to 2.515)	0.841	0.527 (0.150 to 1.853)	0.318

K-DST, Korean developmental assessment of infants; SNP, single nucleotide polymorphism; OXTR, oxytocin receptor; GRIN2B, glutamate ionotropic receptor N-methyl-D-aspartate type subunit 2B; COMT, catechol-O-methyltransferase; PMA, postmenstrual age. B is unstandardized beta.
*Adjusted for gestational age and sex

Supplementary table 3. Associations between OXTR SNPs and K-DST developmental domains in the full-term group

Gene	SNP Name	K-DST* (n=22)											
		Any domain		Gross motor		Fine motor		Cognitive		Language		Sociality	
		Odd ratio (95% CI)	*p*	Odd ratio (95% CI)	*p*	Odd ratio (95% CI)	*p*	Odd ratio (95% CI)	*p*	Odd ratio (95% CI)	*p*	Odd ratio (95% CI)	*p*
OXTR	rs1042778	NA		NA		NA		NA		NA		0.045 (0 to 8.574)	0.248
OXTR	rs2268490	NA		NA		NA		NA		NA		NA	
OXTR	rs2268493	1.553 (0.186 to 13.00)	0.685	NA		NA		NA		NA		NA	

K-DST, Korean developmental assessment of infants; SNP, single nucleotide polymorphism; OXTR, oxytocin receptor; PMA, postmenstrual age. B is unstandardized beta.

***Adjusted for gestational age and sex.**

Supplementary Table 4. Association of allele frequencies with brain volume in preterm infants

Gene	SNP Name	Brain volume (n=59)													
		**White matter**		**Grey matter**		**Deep grey matter**		**Hippocampus**		**Amygdala**		**Cerebellum**		**Brainstem**	
		**B (95% CI)**	***p***	**B (95% CI)**	***p***	**B (95% CI)**	***p***	**B (95% CI)**	***p***	**B (95% CI)**	***p***	**B (95% CI)**	***p***	**B (95% CI)**	***p***
OXTR	rs1042778	-0.274 (-1.964 to 1.416)	0.752	0.667 (-2.115 to 3.449)	0.640	-0.054 (-0.822 to 0.715)	0.891	-0.046 (-0.526 to 0.435)	0.854	-0.191 (-0.971 to 0.589)	0.633	-0.096 (-0.522 to 0.330)	0.660	-0.007 (-0.185 to 0.172)	0.943
OXTR	rs2268490	1.067 (-0.353 to 2.487)	0.147	-0.323 (-3.803 to 0.923)	0.771	0.133 (-0.525 to 0.791)	0.693	0.075 (-0.336 to 0.486)	0.722	0.201 (-0.466 to 0.867)	0.558	-0.074 (-0.445 to 0.296)	0.695	0.039 (-0.114 to 0.191)	0.622
OXTR	rs2268493	0.829 (-0.458 to 2.117)	0.212	-1.44 (-2.481 to 1.835)	0.238	-0.211 (-0.803 to 0.380)	0.487	-0.138 (-0.507 to 0.232)	0.468	-0.221 (-0.821 to 0.380)	0.475	0.062 (-0.272 to 0.396)	0.718	0.001 (-0.137 to 0.139)	0.991
GRIN2B	rs2268116	-0.568 (-1.788 to 0.652)	0.366	1.674 (-0.307 to 3.655)	0.104	-0.337 (-0.889 to 0.215)	0.236	-0.199 (-0.544 to 0.146)	0.264	-0.359 (-0.918 to 0.201)	0.215	-0.152 (-0.464 to 0.160)	0.839	-0.059 (-0.188 to 0.069)	0.370
GRIN2B	rs2284411	-0.946 (-2.229 to 0.337)	0.154	1.561 (-0.559 to 3.682)	0.155	-0.150 (-0.744 to 0.443)	0.622	-0.138 (-0.507 to 0.232)	0.468	-0.245 (-0.846 to 0.355)	0.427	-0.035 (-0.370 to 0.300)	0.839	-0.047 (-0.184 to 0.090)	0.507
COMT	rs174690	-0.225 (-1.459 to 1.009)	0.722	0.945 (-0.081 to 2.971)	0.365	-0.206 (-0.765 to 0.353)	0.473	-0.171 (-0.519 to 0.177)	0.340	-0.263 (-0.829 to 0.303)	0.367	-0.009 (-0.326 to 0.307)	0.954	-0.071 (-0.199 to 0.058)	0.288
COMT	rs4818	-0.256 (-1.503 to 0.991)	0.689	0.962 (-1.086 to 3.01)	0.361	-0.259 (-0.822 to 0.305)	0.372	-0.151 (-0.504 to 0.201)	0.404	-0.256 (-0.829 to 0.316)	0.384	-0.039 (-0.358 to 0.281)	0.813	-0.001 (-0.132 to 0.131)	0.993
COMT	rs740603	0.511 (-0.709 to 1.731)	0.415	0.347 (-1.68 to 2.374)	0.738	-0.285 (-0.838 to 0.268)	0.317	-0.201 (-0.546 to 0.143)	0.257	-0.307 (-0.868 to 0.253)	0.288	-0.108 (-0.421 to 0.205)	0.503	0.043 (-0.086 to 0.172)	0.514

SNP, single-nucleotide polymorphism; OXTR, oxytocin receptor; GRIN2B, Glutamate ionotropic receptor N-methyl-D-aspartate type subunit 2B; COMT, catechol-o-methyltransferase; SW, small-world; GE, global efficiency; LE, local efficiency; Lp, local path length; PMA, postmenstrual age. B is unstandardized beta.

*Adjusted for gestational age, sex, and PMA

Supplementary Table 5. Clinical characteristics of minor allele frequency of rs2268490 in OXTR

**Characteristics**	**Minor allele group (n =70)**	**Major allele group (n=21)**	***p***
Male sex			
Gestational age, mean ± SD	31.34±3.55	31.24±4.16	0.910
Birth weight, mean ± SD	1739.43±709.53	1667.76±676.39	0.683
Scan age, mean ± SD	38.00±2.35 (44/70)	37.80±1.61 (15/21)	0.762
Moderate to severe BPD	22 (31.4)	8 (38.1)	0.569
Stage II to III ROP	10 (14.3)	4 (19.0)	0.596
**K-DST**			
<-2 SD in any domain	8/66 (12.1)	2 (9.5)	0.745
<-2 SD in gross motor domain	4/66 (6.1)	0 (0)	0.248
<-2 SD in fine motor domain	2/66 (3.0)	0 (0)	0.420
<-2 SD in cognition domain	4/66 (6.1)	1 (4.8)	0.824
<-2 SD in language domain	5/66 (7.6)	0 (0)	0.194
<-2 SD in sociality domain	0 (0)	1 (4.8)	0.075
**BSID-III (30/9)**			
Cognition	101.67±7.92	103.33±9.01	0.594
Language	95.87±6.53	98.00±5.41	0.379
Motor	102.57±11.89	102.44±14.01	0.979
Social-Emotional	105.50±21.75	104.44±11.58	0.850
Adaptive behavior	102.40±15.37	110.56±9.32	0.141
**Brain volume (44/13)**			
White matter	36.37±3.91	35.28±3.92	0.382
Grey matter	47.15±5.02	48.72±3.65	0.301
Deep grey matter	7.00±1.18	6.85±0.29	0.644
Hippocampus	0.87±0.74	0.78±0.09	0.687
Amygdala	0.59±1.20	0.38±0.07	0.535
Cerebellum	6.44±0.68	6.47±0.65	0.901
Brainstem	1.58±0.29	1.52±0.16	0.485
**Brain network (44/15)**			
SW	1.63±0.20	1.77±0.15	**0.015**
GE	0.09±0.02	0.08±0.02	**0.048**
LE	0.14±0.0	0.12±0.03	0.050
Lp	11.28±2.00	13.12±3.29	0.056

Data are expressed as the mean±SD or n (%). SD, standard deviation; OXTR, oxytocin receptor; BPD, bronchopulmonary dysplasia; ROP, retinopathy of prematurity; K-DST, Korean developmental assessment of infants; BSID, Bayley scales of infant & toddler development SW, small-world; GE, global efficiency; LE, local efficiency; Lp, local path length

Supplementary Table 6. Simple effect analysis of network metrics on language score by OXTR rs2268490 allele group

	Group	B	SE	95% CI	*p*
GE	Major allele	29.987	239.548	-1000.705 to 1060.678	0.912
	Minor allele	59.167	107.659	-167.975 to 286.308	0.590
LE	Major allele	24.879	118.612	-485.468 to 535.226	0.853
	Minor allele	4.062	68.060	-139.532 to 147.657	0.953
LP	Major allele	0.092	1.733	-7.366 to 7.551	0.962
	Minor allele	-0.432	0.775	-2.067 to 1.204	0.585
SW	Major allele	20.650	49.849	-193.832 to 235.132	0.719
	Minor allele	-19.079	7.931	-35.812 to -2.346	**0.028**

All models were adjusted for age, sex, and postmenstrual age. B; unstandardized regression coefficient; SE, standard error; CI, confidence interval; df, degrees of freedom.

Supplementary Table 7. Clinical characteristics of minor allele frequency of rs4818 in COMT

**Characteristics**	**Minor allele group (n =42)**	**Major allele group (n=49)**	***p***
Male sex			
Gestational age, mean ± SD	31.38±3.70	31.27±3.70	0.882
Birth weight, mean ± SD	1723.21±721.94	1722.61±686.18	0.997
Scan age, mean ± SD	38.00±2.43 (24/42)	37.91±2.02 (35/49)	0.882
Moderate to severe BPD	16 (38.1)	14 (28.6)	0.335
Stage II to III ROP	7 (16.7)	7 (14.3)	0.754
**K-DST (41/46)**			
<-2 SD in any domain	6 (14.6)	4 (8.7)	0.386
<-2 SD in gross motor domain	3 (7.3)	1 (2.2)	0.253
<-2 SD in fine motor domain	2 (4.9)	0 (0)	0.130
<-2 SD in cognition domain	3 (7.3)	2 (4.3)	0.553
<-2 SD in language domain	4 (9.8)	1 (2.2)	0.129
<-2 SD in sociality domain	0 (0)	1 (2.2)	0.342
**BSID-III (17/22)**			
Cognition	102.06±9.36	102.05±7.18	0.996
Language	96.76±5.48	96.05±6.95	0.728
Motor	101.35±12.04	103.45±12.55	0.601
Social-Emotional	101.18±22.81	108.41±16.86	0.262
Adaptive behavior	99.65±14.51	107.86±13.78	0.079
**Brain volume (22/35)**			
White matter	35.75±3.93	36.36±3.93	0.568
Grey matter	48.33±3.89	46.99±5.22	0.302
Deep grey matter	6.80±0.29	7.07±1.31	0.355
Hippocampus	0.75±0.07	0.91±0.82	0.392
Amygdala	0.39±0.04	0.64±1.13	0.379
Cerebellum	6.42±0.46	6.47±0.78	0.801
Brainstem	1.56±0.24	1.57±0.28	0.778
**Brain network (24/35)**			
SW	1.66±0.20	1.67±0.19	0.899
GE	0.09±0.02	0.09±0.02	0.746
LE	0.14±0.03	0.14±0.03	0.822
Lp	12.02±3.00	11.57±2.11	0.500

Data are expressed as the mean±SD or n (%). SD, standard deviation; COMT, catechol-O-methyltransferase; BPD, bronchopulmonary dysplasia; ROP, retinopathy of prematurity; K-DST, Korean developmental assessment of infants; BSID, Bayley scales of infant & toddler development SW, small-world; GE, global efficiency; LE, local efficiency; Lp, local path length
